# GAL and F2R as immune diagnostic biomarkers for fetal growth restriction

**DOI:** 10.1016/j.isci.2026.115228

**Published:** 2026-03-04

**Authors:** Shiying Chen, Yumin Ke, Zhimei Zhou, Yajing Xie, Weihong Chen, Li Huang, Liying Sheng, Yueli Wang, Binbin Chen, Congmei Yang, Zhuna Wu

**Affiliations:** 1 Department of Gynecology and Obstetrics, The Second Affiliated Hospital of Fujian Medical University, Quanzhou, Fujian 362000, China

**Keywords:** Health sciences, Medicine, Medical specialty, Pediatrics, Reproductive medicine

## Abstract

Fetal growth restriction (FGR) is associated with adverse perinatal outcomes, but reliable diagnostic biomarkers are lacking. Through bioinformatics analysis of public datasets and clinical validation, we identified GAL and F2R as potential immune-related biomarkers associated with FGR. Both genes were consistently upregulated in FGR tissues and correlated with altered immune cell infiltration. These findings suggest that GAL and F2R may serve as diagnostic markers and potential therapeutic targets for FGR, facilitating early intervention and improved neonatal outcomes.

## Highlights

• GAL and F2R are identified as potential immune biomarkers for fetal growth restriction
• Bioinformatics analysis reveals altered immune cell infiltration in FGR placentas
• Machine learning algorithms validate diagnostic efficacy of GAL and F2R
• Clinical validation confirms upregulation of GAL and F2R in FGR tissues

## Graphical abstract

**Figure:**
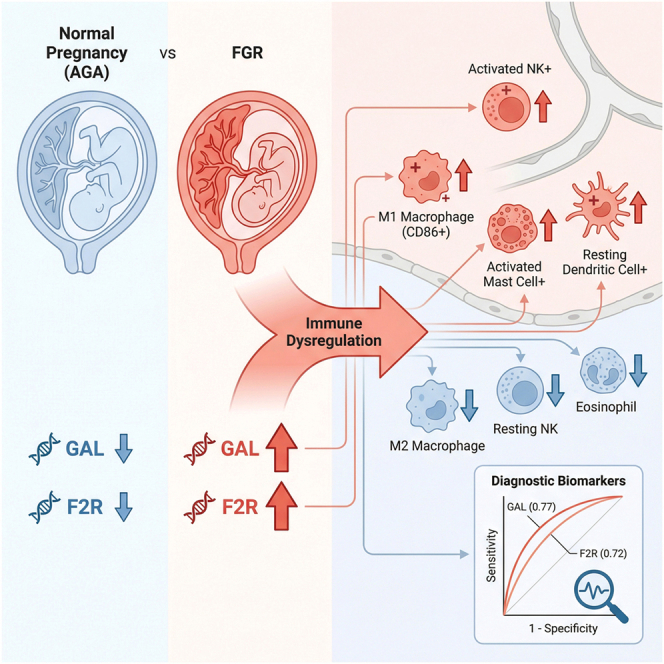

## Introduction

Fetal growth restriction (FGR) refers to the situation where the fetus fails to reach its expected growth potential *in utero*. As a result of one or more maternal, fetal, or placental factors, it is one of the main causes of stillbirth and neonatal death, and has a significant negative impact on the development of multiple systems in the short and long term of the newborn.^1^ Compared with appropriate for gestational age (AGA) infants, FGR induces deviations in the development trajectories of gray and white matter in the brain, reduces the total brain volume and gray matter content of infants, and causes significant differences in electroencephalogram (EEG) activity. These effects persist from birth through childhood and are closely related to adverse neurodevelopmental outcomes such as language delay, cerebral palsy, learning and memory deficits, and behavioral problems.^2^^,^^3^ The long-term risks of cardiovascular disease, impaired bone growth, and muscle cell dysfunction are significantly increased in FGR offspring, although they can achieve catch-up growth through nutritional diets after birth.^4^ In addition, FGR has been confirmed to affect fetal metabolism and endocrine regulation through multiple pathways and mechanisms, thereby promoting the development of obesity, insulin resistance, and other metabolic syndromes in their offspring.^5^

At present, the accurate diagnosis of FGR still poses certain difficulties. In most regions, it is still estimated that fetuses with a weight or abdominal circumference lower than the 10th percentile of the same gestational age are considered high-risk groups for FGR by referring to the statistical data of fetal size in the specific population of that region. However, among them, there are still some healthy small-for-gestational-age infants who only have smaller physical and weight development without structural or functional abnormalities. Additionally, some FGR fetuses may also be missed due to measurement errors, with their estimated weight possibly within the normal range (10th to 90th percentile) for the corresponding gestational age and thus not identified early. Therefore, the accuracy of this standard for predicting FGR is rather limited.^6^

In recent years, research on the correlation between the immune microenvironment and normal and pathological pregnancies has become increasingly mature. Maternal-fetal immune interactions have always been one of the focuses of pathologic obstetrics and reproductive immunology. Innate immune cells such as the complement system, neutrophils, macrophages, dendritic cells, B1 cells, and lymphocytes all regulate the occurrence and development of normal pregnancies through different pathways. The disorder and imbalance of the immune system are considered as one of the important factors leading to adverse pregnancy outcomes.^7^^,^^8^

Given that there are currently no specific molecular markers used for the prediction of FGR and no definite effective treatment methods for FGR, the aim of this study is to identify immune-related molecules associated with FGR diagnosis to improve the diagnostic accuracy of FGR, enable early intervention, and thereby reduce adverse perinatal outcomes such as stillbirth and neonatal mortality. At the same time, it is also hoped that potential new therapeutic targets can be proposed for this group of people to reduce the risk of related complications and improve the poor prognosis of newborns.

## Results

### Study procedure

**Figure 1:**
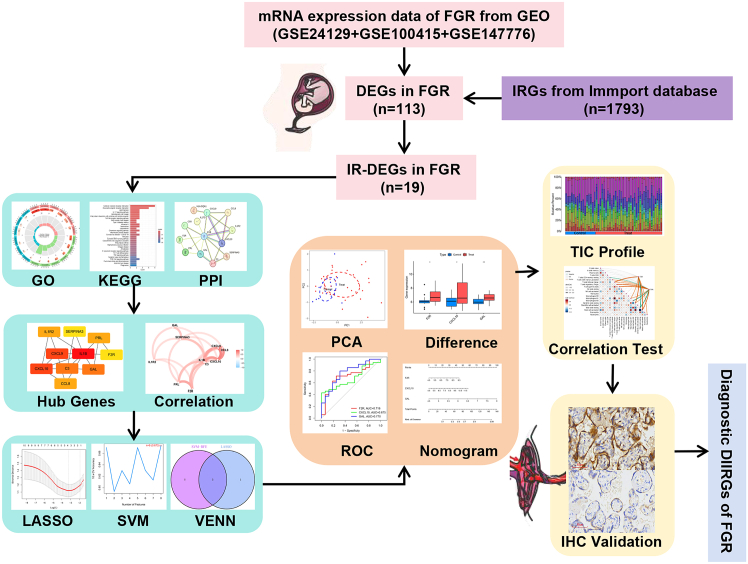
Schematic flowchart of the entire study framework

Figure 1 illustrates the analytical workflow employed in this study. Researchers retrieved microarray data from the Gene Expression Omnibus (GEO) database, and then mapped microarray probes to corresponding gene symbols across each dataset using probe annotation files. By intersecting differentially expressed genes (DEGs) with Immune-related genes (IRGs), they identified Immune-related differentially expressed genes (IR-DEGs). Subsequently, they performed enrichment analyses on these IR-DEGs utilizing the Gene Ontology (GO) and Kyoto Encyclopedia of Genes and Genomes (KEGG) databases. To further screen candidate overlapping genes, they constructed protein-protein interaction (PPI) networks and applied two machine learning algorithms—Least absolute shrinkage and selection operator (LASSO) and support vector machine-recursive feature elimination (SVM-RFE). The predictive performance of the identified biomarkers was assessed through principal component analysis (PCA) and receiver operating characteristic (ROC) curves. The Cibersort algorithm was used to quantify the distribution patterns of 22 immune cell types in FGR, after which correlations between these immune cells and diagnostic biomarkers were analyzed. Finally, immunohistochemical (IHC) staining was carried out on paraffin-embedded specimens that met the inclusion criteria to confirm the study’s findings.

### Identification of IR-DEGs in FGR

**Figure 2:**
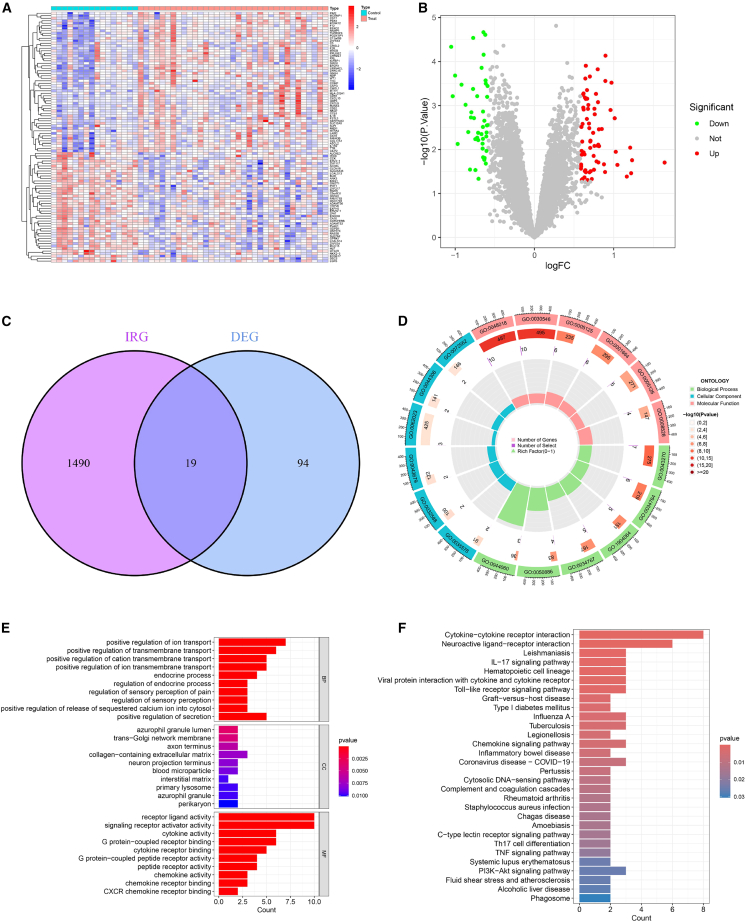
Investigation of DIRG detection and functional properties in the comparison between FGR and AGA (A) Heatmap illustrating expression patterns of top 100 DEGs in the FGR. Each row corresponds to a single gene (with gene names provided in row annotations), and each column represents an individual sample (sample IDs are not displayed). The color gradient is based on row *Z* score normalized expression values: blue denotes low expression levels, while red indicates high expression. (B) Volcano plot presenting the distribution of DEGs. The *x* axis represents log2 fold change (logFC), and the *y* axis denotes −log10(*p* value). Red dots stand for significantly upregulated genes (*p* < 0.05, positive logFC), which exhibit higher expression in FGR; green dots represent significantly downregulated genes (*p* < 0.05, negative logFC), with lower expression in FGR; gray dots indicate non-significant genes. (C) Venn diagram employed to identify intersecting IR-DEGs between IRGs and DEGs. (D) Circular visualization of enriched GO terms. Enriched terms are categorized into three ontologies: BP (outer sector), CC (middle sector), and MF (inner sector). The height of each bar corresponds to the number of genes annotated to a specific term (radial scale range: 0–400), and bar color reflects the enrichment significance (−log10(*p* value); gradient from light to dark indicates increasing significance). Inner points and connecting lines represent the Rich Factor (range: 0–1), a metric reflecting the specificity of each term. GO term IDs are labeled for key enriched terms. (E) Bar plot displaying enriched GO terms. For each GO ontology—BP (top), CC (middle), and MF (bottom)—the top 10 significantly enriched terms (*p* < 0.05) are presented. The *x* axis shows the Gene Ratio (calculated as the number of enriched genes in a term divided by the total number of background genes for that term). (F) Bar plot of enriched KEGG pathways. The top 30 significantly enriched KEGG pathways (*p* < 0.05) are included. The *x* axis represents the Gene Ratio (defined as the number of enriched genes in a pathway divided by the total number of background genes for that pathway).

By comparing 35 FGR samples with 16 AGA samples across the GSE24129, GSE100415, and GSE147776 datasets, our analysis revealed a total of 113 DEGs (Table S1), a heatmap was constructed to offer a visual representation of the top 100 DEGs (Figure 2A). A total of 44 genes were found to be significantly downregulated, whereas the number of genes with significant upregulation was relatively higher, accounting for 69 (Figure 2B). As depicted in Figure 2C, 19 IR-DEGs were identified through the intersection of the DEG list with IRGs (Table S2).

Table S1. 113 DEGs between FGR and AGA patients within the three datasets (GSE24129+GSE100415+GSE147776) This table is provided as a separate Excel file.

### Functional enrichment analysis

Functional enrichment analysis was conducted to explore the biological roles of the 19 IR-DEGs. GO analysis indicated that these genes were primarily enriched in the collagen-containing extracellular matrix and involved in key biological processes such as the positive regulation of ion transport and transmembrane transport. Additionally, they were significantly associated with molecular functions including receptor ligand activity, cytokine activity, and G protein-coupled receptor binding (Figures 2D, 2E, and Data S1). KEGG pathway analysis further revealed significant enrichment in multiple signaling pathways, such as cytokine-cytokine receptor interaction, neuroactive ligand-receptor interaction, leishmaniasis, IL-17 signaling pathway, hematopoietic cell lineage, viral protein interaction with cytokine and cytokine receptor, and toll-like receptor signaling pathway (Figure 2F and Data S1). These results provide valuable insights into the potential biological mechanisms involved in the pathogenesis of FGR, supporting future mechanistic investigations.

Data S1. GO and KEGG enrichment analysis results, related to Figure 2 The file contains the full lists of significantly enriched GO terms (supplemental S1.txt) and KEGG pathways (supplemental S2.txt). Provided as two separate text files.

Data S2. Original immunohistochemistry (IHC) staining images for F2R and GAL, related to Figure 7 Uncropped, full-resolution images of all IHC stains shown in Figure 7, including both low- and high-magnification fields for FGR and AGA samples. Provided as a single PDF file.

### PPI network construction and hub gene selection

**Figure 3:**
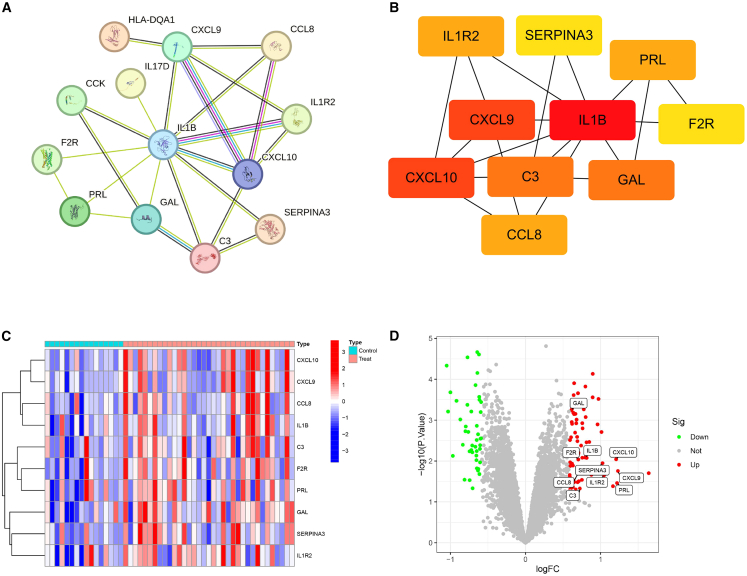
Association characterization between IR-DEGs and hub genes (A) PPI network of 13 IR-DEGs established via the STRING database. Nodes in the network represent proteins, with color coding to distinguish functional clusters: proteins involved in analogous biological roles share identical colors. Lines (referred to as edges) denote predicted functional associations, and edge colors correspond to different types of supporting evidence. The thickness of each line reflects the confidence score of the corresponding interaction. (B) Top 10 hub genes identified using the MCC algorithm via the cytoHubba plugin in Cytoscape. The intensity of node color is proportional to the MCC score: warmer (redder) colors indicate higher centrality scores, signifying greater functional importance of the gene within the PPI network. (C) Heatmap depicting expression patterns of the top 10 hub IR-DEGs in the FGR. (D) Volcano plot illustrating the distribution of the top 10 hub IR-DEGs (with statistical metrics consistent with the DEGs volcano plot in (B), i.e., *x* axis representing log2 fold change [logFC] and *y* axis denoting −log10(adjusted *p* value), to enable direct comparison of hub gene expression differences between groups).

The PPI network of the 19 IR-DEGs was analyzed using the STRING database, followed by the removal of disconnected nodes to generate a refined interaction map. This analysis revealed a connected network comprising thirteen genes: HLA-DQA1, CXCL9, CCL8, IL-17D, CCK, IL1B, IL1R2, F2R, CXCL10, PRL, GAL, C3, and SERPINA3 (Figure 3A). To identify key functional modules, the cytoHubba plugin in Cytoscape was utilized for topological analysis and clustering of the network. Consequently, 10 central nodes forming the maximal clique centrality (MCC) module were identified and clustered (Figure 3B). Notably, all these hub genes—CXCL9, CCL8, IL1B, IL1R2, F2R, CXCL10, PRL, GAL, C3, and SERPINA3—showed significantly upregulated expression levels in the FGR group compared to controls (Figures 3C and 3D).

### Correlation of prospective biomarkers

**Figure 4:**
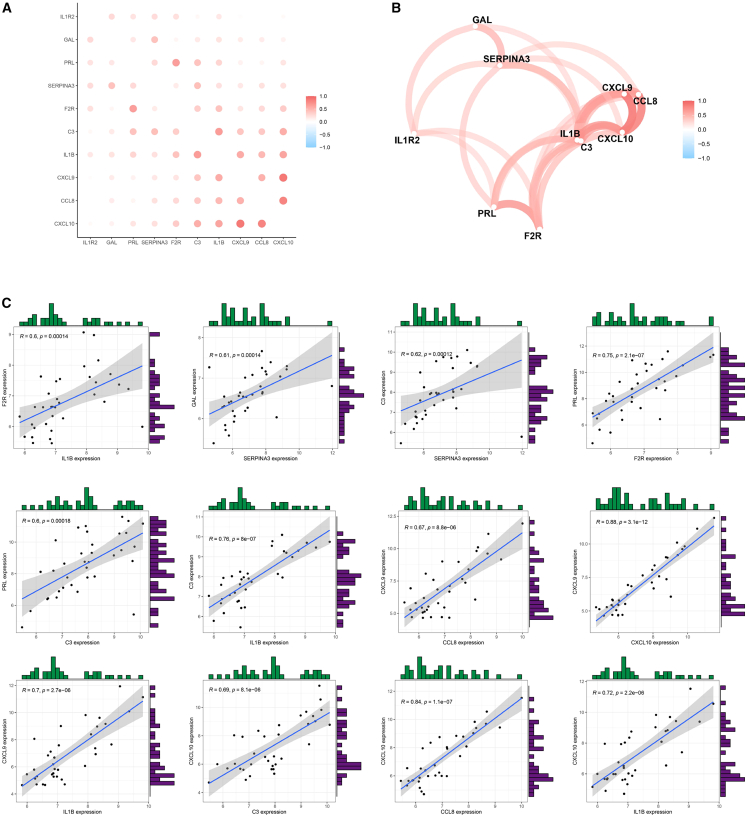
Analysis of correlative patterns among IR-DEGs (A) This heatmap visualizes pairwise Spearman correlations between 10 hub genes. Hierarchical clustering reorders genes to group similar correlation patterns. Red indicates positive correlations, blue indicates negative correlations, and white denotes weak/no correlation. (B) Nodes represent hub genes; edges (colored lines) indicate statistically significant correlations. Edge colors reflect correlation direction: red for positive correlations, blue for negative correlations. Node size and label font were enlarged for visibility. (C) A scatterplot for some highly correlated IR-DEGs is provided.

A correlation analysis was performed among the IR-DEGs to investigate potential relationships in their expression patterns, using a threshold of 0.4 for correlation strength. To visualize the results, the “tidyverse” and “corrr” packages in R were utilized to construct a co-expression correlation heatmap (Figure 4A) and a co-expression network diagram (Figure 4B). Additionally, scatterplots were generated for the 10 gene pairs showing the strongest associations, defined by a correlation coefficient ≥ 0.6 and a statistically significant *p* value < 0.001, in FGR cases (Figure 4C). The results demonstrated that CXCL10 expression in FGR patients was significantly positively correlated with C3, CCL8, CXCL9, and IL1B. C3 also showed strong positive correlations with IL1B (R = 0.76) and SERPINA3 (R = 0.62). Moreover, notable positive associations were observed between F2R and PRL (R = 0.75), IL1B and F2R (R = 0.6), as well as GAL and SERPINA3 (R = 0.61).

### Verification of machine algorithms for diagnostic biomarkers

**Figure 5:**
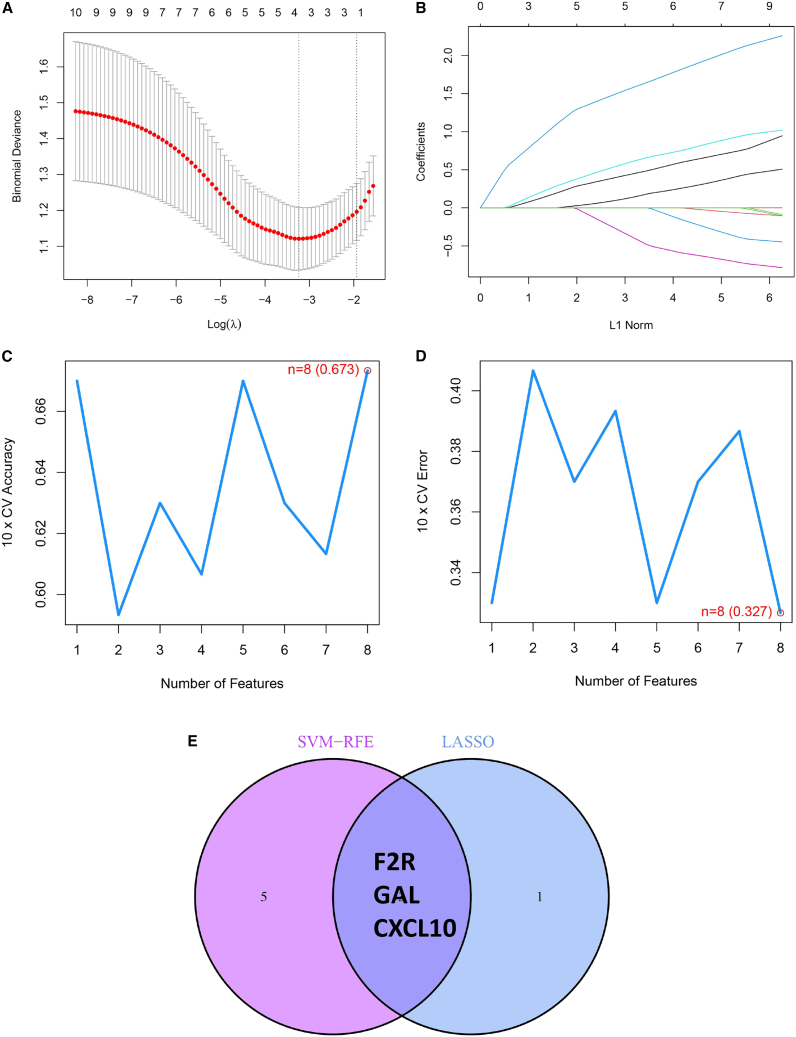
Formulation of a prediction model applied to FGR (A) LASSO regression coefficient profiles of the 4 IR-DEGs are depicted as individual curves, where each curve illustrates the coefficient trajectory of a specific gene across varying regularization parameters (expressed as logλ). (B) A LASSO Cox regression model was employed to generate a plot illustrating the relationship between partial likelihood deviance and log(λ) values. (C) When the cluster number (*k*) was set to 8, the curve representing the total within-cluster sum of squared errors reached the characteristic “elbow point”—a key inflection where further increases in *k* yield minimal reductions in error. (D) At a cluster number (*k*) of 8, the curve corresponding to the average silhouette width (a metric for evaluating cluster compactness and separation) attained its maximum value, indicating optimal clustering performance. (E) A Venn diagram is used to visualize the 3 diagnostic biomarkers that are commonly identified by both the LASSO and SVM-RFE algorithms.

To identify the most significant diagnostic biomarkers for FGR, two machine learning algorithms—LASSO regression and mSVM-RFE—were applied. As shown in Figures 5A and 5B, the LASSO algorithm selected four IR-DEGs from an initial set of ten associated with FGR, specifically: F2R, GAL, CXCL10, and SERPINA3. Meanwhile, mSVM-RFE analysis narrowed down the same set of 10 genes to eight key candidates—F2R, GAL, CXCL10, CXCL9, IL1B, C3, IL1R2, and PRL—with optimal predictive performance, as illustrated in Figures 5C and 5D. By integrating the results from both methods, three overlapping genes—F2R, GAL, and CXCL10—were consistently identified as top-ranking biomarkers, forming the final panel of candidate diagnostic markers for FGR (Figure 5E).

### Effectiveness of diagnostic biomarkers in FGR

**Figure 6:**
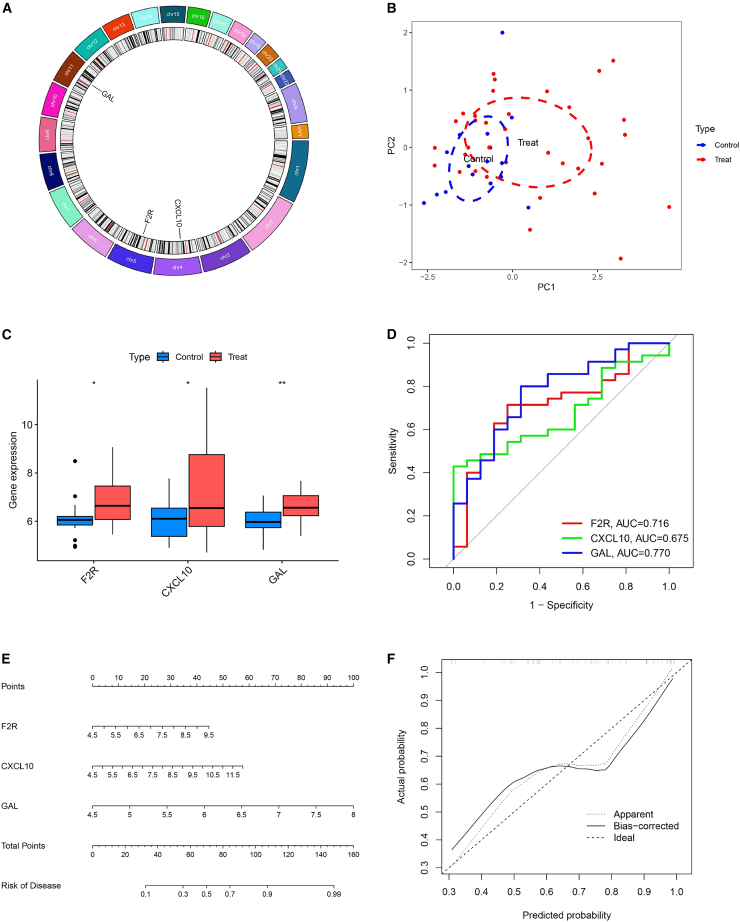
Targeted additional analysis of the three key IR-DEGs (A) Chromosomal positions of the key IR-DEGs are presented. (B) A PCA plot illustrates the distribution of samples based on the expression profiles of the 3 key IR-DEGs. The *x* axis and *y* axis correspond to the first two principal components (PC1 and PC2), respectively, and the percentage of total variance explained by each component is indicated in parentheses adjacent to the axis labels. (C) Comparative expression levels of three crucial IR-DEGs in FGR, which integrates datasets GSE24129, GSE100415, and GSE147776. (D) ROC curves were used to validate the efficacy of three crucial IR-DEGs in predicting FGR, quantifying the diagnostic performance of each gene for FGR identification. (E) A nomogram for predicting the risk of FGR is constructed based on three IR-DEGs, specifically F2R, GAL, and CXCL10. For each of these genes, a corresponding point value is assigned according to its expression level; the total point score is calculated by summing the individual gene points, and this total score is further converted to the predicted risk of developing FGR. (F) A calibration curve for the nomogram is shown, comparing the nomogram-predicted risk of FGR (*x* axis) with the actually observed risk (*y* axis). The diagonal line represents an ideal prediction scenario where predicted and observed risks are identical. The dashed line (“Apparent”) denotes the model’s performance before bias correction, while the solid line (“Bias-corrected”) represents performance after bias correction.

Figure 6A illustrates the chromosomal localization of the three candidate genes—F2R, GAL, and CXCL10. As shown in Figure 6B, PCA results demonstrate that these genes collectively contribute to a clear separation between the FGR and AGA groups, highlighting their potential utility as diagnostic markers for FGR. All three genes exhibited upregulated expression levels in the FGR group compared to controls (Figure 6C). To evaluate their predictive performance, ROC curve analysis was conducted. The AUC values were 0.716 for F2R, 0.770 for GAL, and 0.675 for CXCL10, indicating that F2R and GAL possess stronger discriminatory ability for FGR than CXCL10 (Figure 6D). A nomogram was subsequently constructed to quantify the diagnostic accuracy and predictive potential of these key IR-DEGs (Figures 6E and 6F), integrating their expression levels into a clinically applicable risk assessment model. This tool provides a visual and quantitative means to estimate the likelihood of FGR, facilitating the differentiation between FGR and AGA cases based on the combined gene signature.

### GAL and F2R are both associated with immune infiltration in FGR

**Figure 7:**
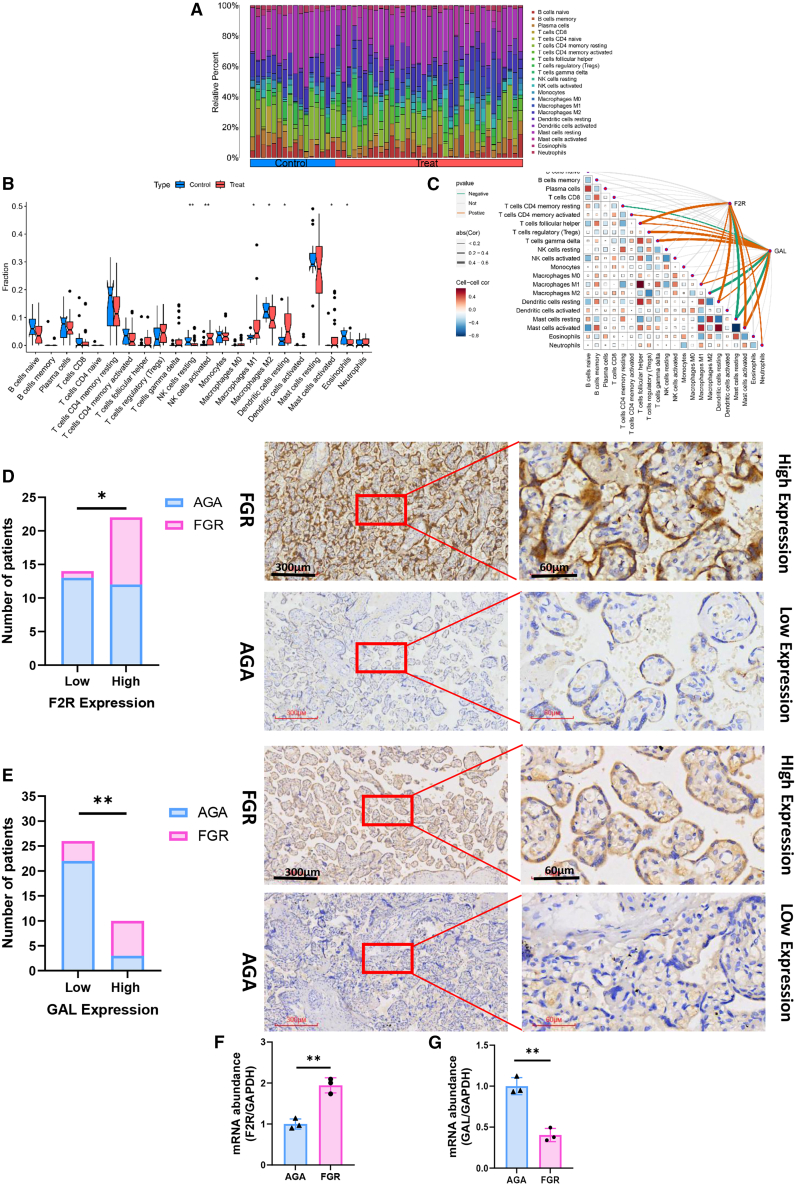
An evaluation focusing on immune infiltration related to two IR-DEGs (A) Stacked bar plot showing the relative abundance of 22 immune cell subtype proportions between FGR and AGA samples. (B) A boxplot is employed to visualize the differentiation in ratios of 22 immune cell types, with a specific focus on comparisons between FGR and AGA. (C) A Spearman correlation network is constructed to illustrate the correlative relationships between two IR-DEGs (namely GAL and F2R) and infiltrating immune cells in FGR. This network visualization explicitly displays how each of the four target IR-DEGs correlates with the 22 types of infiltrating immune cells, facilitating intuitive recognition of positive or negative correlation patterns between the genes and immune cell subsets. (D) Expression levels of F2R are significantly higher in FGR tissues (*n* = 11) compared to AGA samples (*n* = 25). Data are presented as mean ± SEM. (E) Expression levels of GAL are significantly elevated in FGR tissues (*n* = 11) relative to AGA specimens (*n* = 25). Data are presented as mean ± SEM. (F) qRT-PCR analysis demonstrates that F2R mRNA expression is significantly upregulated in FGR placental tissues compared to AGA controls (*p* = 0.0019). Data are presented as mean ± SEM. (G) qRT-PCR analysis reveals a significant downregulation of GAL mRNA in FGR placental tissues compared to AGA controls (*p* = 0.0015). Data are presented as mean ± SEM. Additionally, representative images of IHC staining for F2R and GAL in FGR and AGA patients are presented, illustrating both high and low expression levels of the two genes. All staining images are shown at magnifications of ×40 and ×200, with scale bars clearly indicated for reference. Statistical *p* values were calculated via the chi-square test, where ∗*p* < 0.05 and ∗∗*p* < 0.01.

The CIBERSORT algorithm was utilized to estimate the relative abundances of 22 distinct immune cell types in both FGR and AGA samples (Figure 7A and Table S3), followed by a comparative analysis of immune cell composition between the two groups. The results revealed that the FGR group exhibited significantly higher infiltration levels of activated NK cells, M1 macrophages, resting dendritic cells, and activated mast cells, while showing significantly lower proportions of resting NK cells, M2 macrophages, and eosinophils (Figure 7B). Furthermore, we explored the correlation between the identified diagnostic biomarkers and various tumor-infiltrating immune cells. Expression of F2R was positively associated with follicular helper T cells, M1 macrophages, resting dendritic cells, activated mast cells, and neutrophils, but negatively correlated with resting mast cells. Similarly, GAL expression showed positive correlations with activated CD4^+^ memory T cells, follicular helper T cells, regulatory T cells (Tregs), gamma delta (γδ) T cells, M1 macrophages, resting dendritic cells, and activated mast cells, while it was negatively correlated with resting CD4^+^ memory T cells, M2 macrophages, and resting mast cells (*p* < 0.05) (Figure 7C and Table S4). These findings highlight the strong association of F2R and GAL with immune cell infiltration and suggest their potential involvement in modulating the immunological microenvironment in FGR.

Table S3. The correlation of FGR and immune cell This table is provided as a separate Excel file.

### Validation of F2R and GAL genes from clinical specimens

To validate our prior bioinformatic findings, we examined the expression of F2R and GAL in FGR and AGA tissues using both IHC and qRT-PCR. Consistent with our previous analysis, F2R protein (IHC, *p* = 0.0150; Figure 7D) and mRNA (qRT-PCR, *p* = 0.0019; Figure 7F) levels were significantly upregulated in FGR compared to AGA controls (*p* < 0.05), thus validating its high expression (Data S2). For GAL, IHC results confirmed its high protein expression in FGR (Figure 7E; *p* = 0.0014), aligning with the initial bioinformatic prediction. However, qRT-PCR analysis revealed a significant downregulation of GAL mRNA in FGR tissues (Figure 7G; *p* = 0.0015), presenting a discordant result at the transcriptional level.

## Discussion

FGR has been confirmed to be closely related to long-term neurological, cardiovascular, and other multi-system functional disorders in newborns. However, the current treatment methods for FGR are extremely limited, including maternal nutritional support, rest, oxygen inhalation, and the application of aspirin, etc. But none of them have been proven by evidence to have definite therapeutic effects on the disease.^6^

It is worth noting that in recent years, many scholars have conducted corresponding research on the adverse prognostic genes related to FGR and targeted therapy, and have achieved certain results. Bernardo J. Krause et al. treated FGR pregnancies with N-acetylcysteine (NAC), a precursor of H2S, which increased the expression of the nitric oxide synthase (NOS)/nitric oxide (NO) pathway, thereby improving the function of damaged endothelial cells in the human chorionic artery and effectively preventing the occurrence and development of cardiovascular dysfunction in FGR offspring.^9^ Charlotte Schomig et al. found through research on the brain development of FGR offspring in rats that cell proliferation and mTOR signaling in the hippocampus were dysregulated in different ways depending on the different causes of FGR. By intervening in the expression of mTOR signaling, the long-term neurocognitive function of FGR offspring could be improved.^10^ The normal expression of Jam C and Pard3a in pigs was found to reverse the delayed migration and increased apoptosis of granular cells caused by FGR, which may become a new target for the treatment of FGR-related cerebellar hypoplasia.^11^ However, the majority of these studies remain confined to preclinical or theoretical findings, and the clinical application value is limited.

In addition, with the development of bioinformatics technology, researchers have been constantly identifying and exploring potential diagnostic and therapeutic targets for various diseases through the analysis of large-scale database samples. Currently, some scholars have conducted bioinformatics analyses on FGR and reached certain conclusions, but the number of studies is still relatively limited. Jingjin Yang et al. identified differentially expressed genes in FGR-related GSE datasets and used WGCNA to screen core modules, ultimately determining that CXCL9, CXCR3, and ITGAX could be key genes for FGR. However, they did not conduct further clinical validation, and the possible mechanism of action of the core genes remains unclear.^12^ Xue Wang et al. also screened CTGF as a key gene related to idiopathic FGR through bioinformatics analysis and IHC verification, but the sample size was limited, and the conclusion still needs to be further verified by cell and animal experiments.^13^ Therefore, considering the current research status and adverse outcomes of FGR, as well as the correlation between immune infiltration and pathological obstetrics, we attempt to identify immune-related diagnostic and therapeutic targets in FGR through bioinformatics technology, in order to assist in the early identification of this population. We also hope to explore possible therapeutic targets to achieve catch-up growth in fetal growth retardation and reduce the risk of long-term multi-system complications in newborns.

The factors and molecules involved in the occurrence and development of FGR are complex and diverse. Although there are more and more molecular studies related to FGR, and some of them involve immune-related analyses, the exact pathogenesis and signaling pathways of FGR remain unclear. Zhongmei Yang et al. analyzed the metabolomics of the maternal-fetal interface (including placenta, maternal, and fetal serum) in FGR patients and found that the content of fatty acids in the placenta was significantly increased, while it was significantly decreased in the fetal serum. Further integrated analysis of transcriptomics and metabolomics suggested that it might be related to abnormal signaling pathways of linoleic acid metabolism. Then, they detected and analyzed the expression of molecules potentially related to FGR in the metabolic pathway and found that PLA2G2A and CYP2J2 expression levels were increased in the placenta, while PLA2G4C expression was decreased. This result provides a new perspective for clarifying the potential metabolic molecular mechanism of FGR.^14^ Tai-Ho Hung et al. found that compared with AGA and LGA, the phosphorylation levels of AKT and mTOR in the placenta of FGR pregnant women were significantly decreased, while the phosphorylation level of AMPKα was significantly increased. The use of AKT inhibitors could further reduce the phosphorylation level of mTOR, while AMPK inhibitors could increase the phosphorylation level of mTOR to some extent, although the difference was not statistically significant. This result indicates that AKT and AMPK may be involved in the occurrence of FGR by regulating the activity of mTOR in placental trophoblasts.^15^ Ming-Rui Li established a HMGB1 knockout mouse model through RNAi technology and observed a significant decrease in the weight of the offspring and placenta of the mice, as well as obvious skeletal development damage. Further studies on the changes in autophagy in the placenta found that the specific knockout of HMGB1 disrupted autophagic flux, reduced autophagy levels, and ultimately led to placental developmental abnormalities. The ERK signaling pathway was confirmed to mediate the autophagic regulation of HMGB1 on placental trophoblasts, thereby affecting cell viability, proliferation, and migration.^16^ Overexpression of Mir483 or an increase in the phosphorylation level of insulin-like growth factor binding protein-1 mediated by PKA in the decidua can reduce the bioavailability of IGF-1.^17^^,^^18^ Inactivation of MAP3K4 kinase was also found to lead to a decrease in the expression of insulin-like growth factor 1 receptor (IGF1R) and insulin receptor (IR), as well as a reduction in AKT activation,^19^ all of which can promote the occurrence of FGR. Toll-like receptor 4 plays a crucial role as a receptor in regulating maternal immune adaptation during pregnancy. Its deletion mutation can lead to impaired maternal immune tolerance, thereby causing pregnancy loss or FGR.^20^ In addition, the increased expression of imprinted genes such as PHLDA2, CDKN1C, and PEG10, as well as epigenetic regulators such as DNMT1, DNMT3A, DNMT3B, and TET3, is also considered a potential cause of FGR.^21^ We used IRGS data and DEGS for cross-analysis and obtained 19 overlapping IR-DEGS. GO and KEGG analysis indicated that these DEGS were mainly enriched in cytokine-cytokine receptor interaction pathways, neuroactive ligand-receptor interaction pathways, IL-17 and Toll-like receptor signaling pathways, and had positive regulatory effects on ion transport, receptor-ligand, and cytokine activity. This provides some help for us to further explore the immune regulatory pathways related to FGR.

Our study further confirmed three DEGs through the integration of two machine algorithms and verified their diagnostic efficacy using ROC curves. Ultimately, GAL and F2R were identified as potential biomarkers for FGR diagnosis. As a member of the animal lectin family, GAL is one of the main regulators of cell interactions in the microenvironment and is involved in the occurrence and development of various metabolic diseases and tumors.^22^^,^^23^ GAL-3 gene knockout in the IgA nephropathy model can significantly improve urinary protein and renal function, and reduce the severity of renal pathology, including neutrophil infiltration and the reduction of Th17 cell differentiation in renal draining lymph nodes.^24^ GAL-8 can bind to the immunosuppressive receptor LILRB4, activate STAT3, and inhibit NF-κB to suppress monocyte-like myeloid-derived suppressor cells (M-MDSCs), thereby promoting tumor cell growth.^25^ GAL has also been found to be expressed at the maternal-fetal interface, participating in maternal immune tolerance, angiogenesis, trophoblast invasion, and placental development, playing an important role in embryonic development.^23^^,^^26^ Some studies have suggested that the dysregulation of GAL may be related to the occurrence and development of FGR. The absence of maternal GAL-3 can lead to the dysbiosis of the maternal intestinal microbiota, induce placental inflammation and poor perfusion, as well as the activation and infiltration of uterine natural killer cells, resulting in placental dysfunction and driving the occurrence of FGR.^27^^,^^28^ Xiao-Xiao Jin et al. verified the low expression of GAL-1 in the serum and placenta of FGR mothers, and the level of GAL-1 was positively correlated with birth weight.^29^ These research results further support the correlation between GAL and FGR, suggesting that targeted therapy against GAL may reverse the adverse prognosis related to FGR. Our study observed a significant increase in GAL expression in the FGR group. In the future, we will consider conducting more in-depth research on the role of certain specific members of the GAL family in FGR.

The F2R gene encodes protease-activated receptor-1 (PAR-1), which serves as the primary thrombin receptor on platelets and can bind to various ligands such as thrombin, coagulation factors, and matrix metalloproteinases (MMPs), playing a crucial role in the platelet activation cascade.^30^ F2R has been successively confirmed to be upregulated in multiple malignant tumors including gastric cancer,^31^ glioma,^32^ and breast cancer.^33^ It may activate the β-catenin signaling pathway, participate in cell proliferation and invasion, and be significantly associated with poor prognosis of diseases. The activation of F2R can promote the initiation of the coagulation cascade, especially in vascular damage caused by immune complexes, leading to the progression of SLE. Additionally, F2R has been demonstrated to have the potential to serve as a molecular biomarker for predicting the therapeutic response of SLE patients.^34^ These studies all emphasize that F2R can be a promising diagnostic and prognostic biomarker related to tumors and immune diseases, and may become a potential guiding factor for the development of future-targeted therapies. Currently, some scholars have also revealed the correlation between F2R and pathological obstetrics. PAR-1 is expressed on neutrophils in pregnant patients but not in non-pregnant patients, promoting inflammation through mediating cyclooxygenase-2 (COX-2) and participating in the occurrence of preeclampsia.^35^ Moreover, F2R shows a higher expression level in uterine myocytes during delivery, suggesting that F2R may play an important role in the regulation of uterine myometrial function during preterm birth and delivery.^36^ Currently, there are no research reports on the correlation between F2R and the occurrence and development of FGR. Our research results show that F2R is significantly overexpressed in the FGR group. In the future, we will further explore the potential mechanisms and pathways related to F2R in FGR, which may become one of the emerging targets for the treatment of FGR.

This study experimentally validated the key candidate genes identified through bioinformatics screening using both IHC and qRT-PCR. For F2R, both its protein and mRNA levels showed consistently high expression in FGR tissues, which aligns with and reinforces the preliminary computational analysis, further solidifying the reliability of F2R as a potential immune-related diagnostic biomarker for FGR. In contrast, the expression pattern of GAL revealed a discordance between transcriptional and translational levels: IHC detection confirmed high protein expression in FGR tissues, consistent with the bioinformatic prediction, while qRT-PCR indicated significant downregulation at the mRNA level. This discrepancy may highlight important post-transcriptional regulatory mechanisms involved in the pathogenesis of FGR. For instance, the stability of GAL protein could be enhanced, or its translation efficiency might be specifically upregulated in the FGR state in response to changes in the local placental immune microenvironment. As reviewed, galectin expression and function at the maternal-fetal interface are governed by complex, multi-level regulation.^26^ This discrepancy may reflect altered protein stability, translational efficiency, or isoform-specific regulation under FGR-associated stress, consistent with GAL’s role as a placental immunomodulator.^26^ As noted by Oravecz et al. (2022), placental galectins such as gal-13 and gal-14 undergo intricate multi-level regulation encompassing transcriptional, translational, subcellular localization, and microenvironmental cues.^37^ In pregnancy complications like preeclampsia, their expression patterns can be substantially reprogrammed. In the context of FGR, the increased GAL protein may arise from enhanced protein stability, upregulated translational efficiency, or contributions from locally infiltrating immune cells under stress conditions. Furthermore, galectins often form high-density “glycan lattices” on cell surfaces, where their functional concentration can operate independently of bulk mRNA levels.^37^ Thus, the uncoupled expression of GAL likely reflects altered protein turnover, translational control, or isoform-specific regulation under FGR-associated stress, consistent with its role as a placental immunomodulator.^26^ This transcriptional-translational decoupling may represent a distinctive, disease-specific adaptive mechanism in FGR pathophysiology. While the marked differential expression of GAL at the protein level retains clinical diagnostic value as a potential histological biomarker, further mechanistic studies are needed to define the exact pathways underlying this dissociation. Future investigations employing proteomics, RNA-stability assays, glycomic analyses, and spatial transcriptomics will help elucidate the cellular origins, regulatory networks, and functional implications of GAL in FGR. In summary, whereas F2R shows consistent upregulation from gene to protein, the case of GAL suggests a more complex, layered gene-expression regulatory network active in FGR.

To further understand the immune microenvironment of FGR, we observed through CiberSort analysis that the proportion of activated NK cells, macrophages M1, resting dendritic cells and activated mast cells was significantly higher in FGR. Gurman Kaur et al. demonstrated that the interaction between KIR2DL1 expressed on uterine NK cells and paternally inherited HLA-C∗0501 expressed on fetal trophoblast cells can lead to pathogenic uterine artery remodeling and changes in NK cell function, thereby causing FGR.^38^ Romy E. Bezemer et al. also observed a reduction in M2 macrophages in FGR placental tissue, but a significant increase in the overall proportion of macrophages and Treg cells, which is consistent with our research results.^39^ Additionally, MC can be divided into two subtypes: tryptase-positive (MCT) and tryptase- and chymase-positive (MCTC). Mirthe H. Schoots et al. observed a significantly lower proportion of MCTC in the decidua of FGR, but no significant difference in MCT, suggesting that the relative deficiency of MCTC may be associated with the development of FGR.^40^ These research results all contribute to our deeper functional studies on immune cell subtypes related to FGR and provide guidance for exploring immune-targeted therapies for FGR.

In addition, we further analyzed the correlation between DEGS and the immune microenvironment. The results showed that GAL was positively correlated with activated T cells CD4 memory, T cells follicular helper, Tregs, T cells gamma delta, macrophages M1, resting dendritic cells and activated mast cells, and negatively correlated with resting T cells CD4 memory, macrophages M2 and resting mast cells. Tommy Lidstrom et al. observed in pancreatic ductal adenocarcinoma (PDAC) that after reducing the expression of GAL-4, the proportion of M1 macrophages, T cells and antigen-presenting dendritic cells increased, and the survival time was prolonged, suggesting that GAL-4 is involved in immune evasion during tumor development.^41^ GAL-9, as a negative regulator of adaptive immune response, enhances antitumor immunity by promoting the maturation of CD11c+ DCs and activating CD4^+^ TIM3+ and CD8^+^ TIM3+ T cells.^42^ GAL-13 and GAL-14 are expressed in placental tissue and are believed to promote maternal-fetal immune tolerance by inducing apoptosis of activated T lymphocytes and polarizing neutrophils to an immunoregulatory phenotype.^37^ In the future, we still need to conduct further research on the role of each subtype of GAL in the immune microenvironment of FGR. Additionally, our research results showed that F2R expression was positively correlated with T cells follicular helper, macrophages M1, resting dendritic cells, activated mast cells and neutrophils, while negatively correlated with resting mast cells. Currently, there are relatively few studies on the correlation between F2R and immune cells. As one of the genes related to CD8^+^ T cell infiltration, it has been used in the construction of a prognostic model for adenocarcinoma of the gastroesophageal junction (ACGEJ).^43^ Additionally, F2R is one of the members of the genomic model for predicting the prognosis of low-grade glioma, and its expression level has been confirmed to be significantly negatively correlated with dendritic cells, Th1, and Th2.^44^ Although the number of studies is limited, they all support to some extent that GAL and F2R have potential as immune-related diagnostic markers and therapeutic targets.

In conclusion, through integrated bioinformatics and clinical validation, we identified GAL and F2R as key immune-related molecules implicated in FGR. These findings lay a foundation for improving early diagnosis and targeted intervention, which may help reduce adverse perinatal outcomes and long-term multi-system complications in affected newborns.

### Limitations of the study

This study has limitations. The use of public datasets and a modest clinical sample size limits the generalizability of the findings. Validation in larger, prospective cohorts is needed. The unresolved discrepancy in GAL expression between mRNA and protein levels requires further mechanistic investigation using advanced molecular techniques. Crucially, the functional roles of GAL and F2R in FGR pathogenesis remain speculative and necessitate direct experimental validation in relevant cellular and animal models. Finally, our analysis of the immune microenvironment remains descriptive; deeper mechanistic insights into specific cellular interactions are required. Future studies addressing these limitations will be essential to translate these biomarker discoveries into clinically useful tools for FGR.

## Resource availability

### Lead contact

Further information and requests for resources and reagents should be directed to and will be fulfilled by the lead contact, Zhuna Wu (wuzhuna@aliyun.com).

### Materials availability

This study did not generate new unique reagents.

### Data and code availability

#### Data

The microarray datasets used in this study are available in the GEO database under accession numbers GSE24129, GSE100415, and GSE147776. The original code supporting the findings of this study has been deposited in the Zenodo repository under DOI: https://doi.org/10.5281/zenodo.18137533 (version V1.0.0-alpha).

#### Code

All scripts, algorithms, and computational models used for data analysis are available at Zenodo (DOI: https://doi.org/10.5281/zenodo.18137533).

#### Other

Any additional information required to reanalyze the data reported in this paper is available from the lead contact upon request.

## STAR★Methods

### Key resources table

**Table:** 

REAGENT or RESOURCE	SOURCE	IDENTIFIER
**Antibodies**		

Rabbit anti-GAL (for IHC)	Bioss, Beijing	Cat:bs-0017M; RRID: AB_10855141
Rabbit anti-F2R (for IHC)	Bioss, Beijing	Cat:bs-0828R; RRID: AB_10857704

**Biological samples**		

Human placental tissues (FGR & AGA)	The Second Affiliated Hospital of Fujian Medical University	Ethics Approval: 2024-399

**Chemicals, peptides, and recombinant proteins**		

TRIzol Reagent	Beyotime Biotechnology, China	Cat# R0016
cDNA Synthesis Kit	TaKaRa, Japan	Cat# RR047A

**Deposited data**		

GEO: FGR and AGA expression datasets	NCBI GEO	GSE24129, GSE100415, GSE147776
ImmPort: Immune-related genes	ImmPort Database	https://www.immport.org/shared/
Processed data and code (Zenodo)	This paper	DOI: https://doi.org/10.5281/zenodo.18137533

**Oligonucleotides**		

qPCR primer: GAPDH-F	This paper	Sequence: 5′-GTCTCCTCTGACTTCAACAGCG-3′
qPCR primer: GAPDH-R	This paper	Sequence: 5′-ACCACCCTGTTGCTGTAGCCAA-3′
qPCR primer: GAL-F	This paper	Sequence: 5′-GTCTGTGTGCTGTAACCTGAAGTC-3′
qPCR primer: GAL-R	This paper	Sequence: 5′-GCAAAGAGAACAGGAATGGCT-3′
qPCR primer: F2R-F	This paper	Sequence: 5′-TGGGTCTGAATTGTGTCGCT-3′
qPCR primer: F2R-R	This paper	Sequence: 5′-AGAGTACGCCAGGAGAGGGA-3′

**Software and algorithms**		

R	R Foundation	v4.1.3; https://www.r-project.org/
limma R package	Bioconductor	v3.50.3
ggplot2 R package	CRAN	V4.0.1
clusterProfiler R package	Bioconductor	v4.2.2
glmnet R package	CRAN	v4.1.8
e1071 R package	CRAN	v1.7.16
pROC R package	CRAN	v1.18.5
Cytoscape	Cytoscape Consortium	v3.10.0; https://cytoscape.org/
STRING database	STRING Consortium	v11.5; https://string-db.org/
CIBERSORT algorithm	Newman et al., 2015	https://cibersort.stanford.edu/

**Other**		

IHC scoring protocol	This paper	Described in Methods: Immunohistochemistry
LASSO + SVM-RFE feature selection	This paper	Described in Methods: Screening biomarkers

### Experimental model and study participant details

#### Human participants

• Sample source: Placental tissues
• Sample size: 11 FGR cases, 25 AGA controls
• Sex: Female (pregnant women)
• Ethnicity: Chinese
• Age/Developmental stage: Full-term pregnancies (gestational age determined by last menstrual period and ultrasound)
• Inclusion criteria: FGR defined as estimated fetal weight (EFW) or abdominal circumference (AC) <10th percentile for gestational age according to SMFM Practice Bulletin; AGA defined as birth weight between 10th and 90th percentiles
• Exclusion criteria: Pregnancy complications (e.g., preeclampsia, intrauterine infection), multiple gestation, congenital anomalies, chromosomal abnormalities, maternal autoimmune diseases, pre-gestational diabetes, and clinical chorioamnionitis
• Institutional permission: Ethical approval granted by the Research Ethics Committee of the Second Affiliated Hospital of Fujian Medical University (approval number: 2024-399)
• Informed consent: Written informed consent obtained from all participants

#### Data sources

• Public datasets: GSE24129 (GPL6244, 8 FGR, 8 AGA), GSE100415 (GPL6244, 20 FGR), GSE147776 (GPL20844,7 FGR, 8 AGA) from GEO database (https://www.ncbi.nlm.nih.gov/geo/)
• IRGs source: ImmPort database (https://www.immport.org/shared/)

### Method details

#### Data collection and processing

The training cohort datasets (GSE24129, GSE100415, and GSE147776), comprising 35 FGR and 16 AGA samples, were retrieved from the GEO database (https://www.ncbi.nlm.nih.gov/gds) (Tables 1, S5, and S6). These three datasets were integrated into a unified meta-cohort, and batch effects were corrected using the “SVA” package in R.

**Table 1:** mRNA expression profiles related to FGR and AGA samples

Dataset ID	Platform	FGR	AGA
GSE24129	GPL6244	8	8
GSE100415	GPL6244	20	0
GSE147776	GPL20844	7	8
Total	–	35	16

#### Identification and functional enrichment of IR-DEGs

DEGs were identified using the “limma” package in R, with selection criteria set as |log2 fold change| ≥ 0.585 and P-value <0.05. A heatmap and a volcano plot were generated using the “ggplot2” package in R to visualize the expression patterns and significance of the DEGs. To identify IR-DEGs associated with FGR, an overlap analysis was performed between the DEGs and IRGs retrieved from the ImmPort database (https://www.immport.org/shared/) (Table S7). Functional enrichment analyses, including Gene Ontology (GO) terms—covering biological processes (BP), cellular components (CC), and molecular functions (MF)—and KEGG pathway analysis, were conducted on the IR-DEGs using the R packages “enrichplot”, “org.Hs.e.g.,.db”, “clusterProfiler”, and “DOSE”, with a statistical cutoff of P < 0.05. The results of these enrichment analyses were visualized using the “ggplot2” package.

Table S7. The IRGs from the Import database This table is provided as a separate Excel file.

#### PPI network construction and analysis

The protein-protein interaction (PPI) network was constructed using the STRING database (https://string-db.org/) by inputting the IR-DEGs under the “multiple proteins” option and selecting “Homo sapiens” as the organism. Gene symbols were extracted from protein IDs, and interactions without corresponding gene names were excluded. The filtered PPI data were then imported into Cytoscape 3.10.0 for network visualization. To identify central nodes within the network, the cytoHubba plugin was applied to rank genes based on their topological features. Through this analysis, key genes potentially involved in the pathogenesis of FGR were prioritized.

#### Screening biomarkers for FGR by machine learning algorithms

To identify robust diagnostic biomarkers for FGR, Spearman correlation analysis was employed to screen IR-DEGs with a |log2 fold change (FC)| ≥ 0.585. The selection of key biomarkers was further refined using the Least Absolute Shrinkage and Selection Operator (LASSO) regression method integrated with multiple support vector machine recursive feature elimination (mSVM-RFE), enhancing the accuracy and reliability of biomarker discovery.

The “glmnet” package in R was used to implement LASSO regression, a penalized regression approach designed for feature selection that helps reduce model complexity and prevent overfitting. Concurrently, the mSVM-RFE algorithm was applied using the “e1071” package, which leverages support vector machines within a recursive elimination framework guided by supervised learning. This method enhances feature selection stability through resampling at each iteration, progressively removing less significant features based on SVM-derived weight vectors. Given its improved resistance to overfitting compared to traditional SVM-RFE, mSVM-RFE was integrated with LASSO analysis to identify consistently selected overlapping genes, thereby increasing the robustness of candidate biomarker identification.

Finally, ROC analysis was conducted using the R package “pROC” to evaluate the diagnostic performance of the identified biomarker. The area under the curve (AUC) was calculated to assess its ability to accurately differentiate FGR cases from AGA controls.

#### Establish and validate the nomogram model for assessing diagnostic ability of FGR

Principal Component Analysis (PCA) leverages eigenvalue decomposition to lower the dimensionality of gene expression datasets. It also assesses the statistical significance of inter-group differences by means of grouped confidence ellipses. To further validate the diagnostic potential of IR-DEGs for FGR, PCA was employed with the assistance of the “limma” and “ggplot2” R packages. A predictive nomogram for FGR was developed using the “rms” and “rmda” packages in R. In this model, each predictor variable is assigned a corresponding score labeled as “points,” and the sum of these individual scores yields the “total points,” which reflects the overall predicted probability of FGR. To assess the model’s accuracy, calibration curves were plotted to evaluate the agreement between the predicted and observed outcomes.

#### Evaluating the level of immune infiltration in FGR and biomarkers

The CIBERSORT algorithm (http://cibersort.stanford.edu/) was applied to estimate the relative abundance of 22 tumor-infiltrating immune cell types in the tissue samples. Correlation patterns among these immune populations were analyzed and visualized using the “corrplot” package in R. To examine differences in immune cell infiltration between the FGR and SGA groups, violin plots were generated using the “vioplot” package. Furthermore, Pearson’s correlation analysis was performed to assess the relationships between the identified diagnostic biomarkers and the levels of immune cell infiltration, with results plotted using the “ggplot2” package for enhanced graphical representation.

#### Patient and tissue samples

The study samples were obtained from placental paraffin-embedded tissues of 11 FGR and 25 AGA, collected at the Second Affiliated Hospital of Fujian Medical University between January 2021 and May 2025. FGR was strictly defined according to the SMFM Practice Bulletin:^45^ Estimated fetal weight (EFW) or abdominal circumference (AC) <10th percentile for gestational age. AGA controls were defined as birth weight between the 10th and 90th percentiles. Exclusion criteria included Pregnancy complications (e.g., preeclampsia, intrauterine infection), multiple gestation, congenital anomalies, chromosomal abnormalities, maternal autoimmune diseases, pre-gestational diabetes, and clinical chorioamnionitis. Prior to initiation of the study, ethical approval was granted by the Research Ethics Committee of the Second Affiliated Hospital of Fujian Medical University.

#### Ethical approval and patient information

The research was approved by the Research Ethics Committee of The Second Affiliated Hospital of Fujian Medical University before the study (approval ethics number: 2024-399). Patient sample details: source (placental tissue), number (11 FGR, 25 AGA), sex (female), and ethnicity (Chinese). Confirmation of informed consent. The study is not part of a clinical trial.

#### Immunohistochemistry (IHC)

IHC staining was performed according to previously established protocols using primary antibodies against GAL (Bioss, Beijing, China, catalog number: bs-0017M, RRID: AB_10855141) and F2R (Bioss, Beijing, China, catalog number: bs-0828R, RRID: AB_10857704). Staining intensity grading: Four categories (0-3 points): negative (0), light yellow (1), brownish yellow (2), tan (3). Proportion scoring: <1/3 (1 point), 1/3-2/3 (2 points), >2/3 (3 points). Final score: Intensity × Proportion; cutoff = 6 (low <6, high ≥6). Based on these composite scores, tissue samples were stratified into low-expression and high-expression groups, with a cutoff value of 6: samples scoring below 6 were classified as low expression, while those with scores of 6 or higher were considered high expression.

#### Quantitative real-time PCR (qRT-PCR)

Total RNA was extracted from placental tissues collected immediately after vaginal delivery or cesarean section using TRIzol reagent (Beyotime Biotechnology, China). cDNA was then synthesized according to the manufacturer’s protocol (TaKaRa, Japan). qRT-PCR was performed to assess the mRNA expression levels of GAL and F2R, with GAPDH serving as the internal reference gene. Relative expression was calculated using the 2−ΔΔCT method. All reactions were run in triplicate for each sample, and three independent experimental replicates were conducted. The primer sequences used are as follows:

GAPDH

Forward: 5′-GTCTCCTCTGACTTCAACAGCG-3′

Reverse: 5′-ACCACCCTGTTGCTGTAGCCAA-3′

GAL

Forward: 5′-GTCTGTGTGCTGTAACCTGAAGTC-3′

Reverse: 5′-GCAAAGAGAACAGGAATGGCT-3′

F2R

Forward: 5′-TGGGTCTGAATTGTGTCGCT-3′

Reverse: 5′-AGAGTACGCCAGGAGAGGGA-3′

### Quantification and statistical analysis

• Software: R software (version 4.1.3)
• Statistical tests: Mann-Whitney U test for group comparisons, Spearman correlation analysis, LASSO regression, SVM-RFE, ROC curve analysis, Pearson’s correlation coefficient, unpaired t-tests
• Significance threshold: P < 0.05 (∗p < 0.05, ∗∗p < 0.01)
• Sample size (n):
  Bioinformatics analysis: 35 FGR, 16 AGA samples from three GEO datasets
  Clinical validation: 11 FGR, 25 AGA placental tissue samples
• Data representation: All quantitative data are presented as mean±SEM unless otherwise specified
• Replication: qRT-PCR reactions run in triplicate for each sample, with three independent experimental replicates

## Acknowledgments

The authors acknowledge the 10.13039/100000085GEO database for providing data on FGR available, and the Import database for providing data on immune-related genes (IRGs). This work was supported by the Innovation of Science and Technology, Fujian province (grant number: 2024Y9412), the 10.13039/501100017686Fujian Provincial Health Technology Project (2024GGA044), Joint funds for the Innovation of Science and Technology, Fujian province (2023Y9234), and the Second Affiliated Hospital of Fujian Medical University Doctoral Miaopu Project (BS202401).

## Declaration of interests

The authors declare that they have no competing interests.
