# Supplementary figures and images for: GAL and F2R as immune diagnostic biomarkers for fetal growth restriction
Source: iScience. 2026 Mar 4;29(4):115228. doi: 10.1016/j.isci.2026.115228 (PMC13015252; doi:10.1016/j.isci.2026.115228)

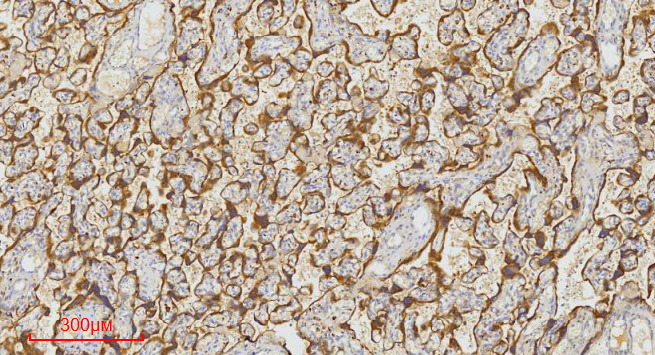

Supplement: Data S2. Original immunohistochemistry (IHC) staining images for F2R and GAL, related to Figure 7 — Uncropped, full-resolution images of all IHC stains shown in Figure 7, including both low- and high-magnification fields for FGR and AGA samples. Provided as a single PDF file. [file mmc6.zip › Data2/(FGR,F2R高表达,低倍镜x4)21020566 6_viewcapture.png]

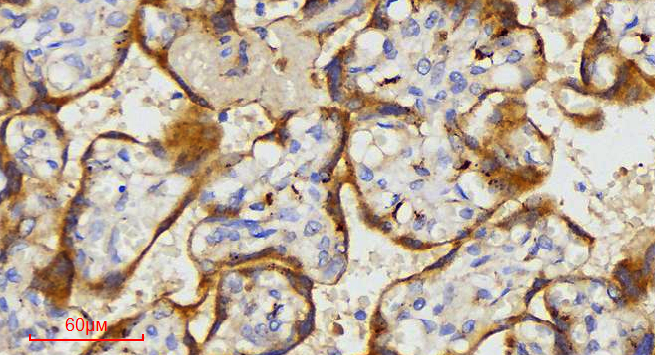

Supplement: Data S2. Original immunohistochemistry (IHC) staining images for F2R and GAL, related to Figure 7 — Uncropped, full-resolution images of all IHC stains shown in Figure 7, including both low- and high-magnification fields for FGR and AGA samples. Provided as a single PDF file. [file mmc6.zip › Data2/(FGR,F2R高表达,高倍镜x20)21020566 6_viewcapture.png]

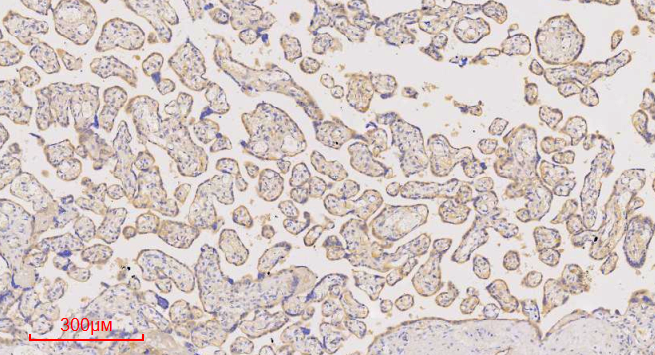

Supplement: Data S2. Original immunohistochemistry (IHC) staining images for F2R and GAL, related to Figure 7 — Uncropped, full-resolution images of all IHC stains shown in Figure 7, including both low- and high-magnification fields for FGR and AGA samples. Provided as a single PDF file. [file mmc6.zip › Data2/(FGR,GAL高表达,低倍镜x4)2132451 5_viewcapture.png]

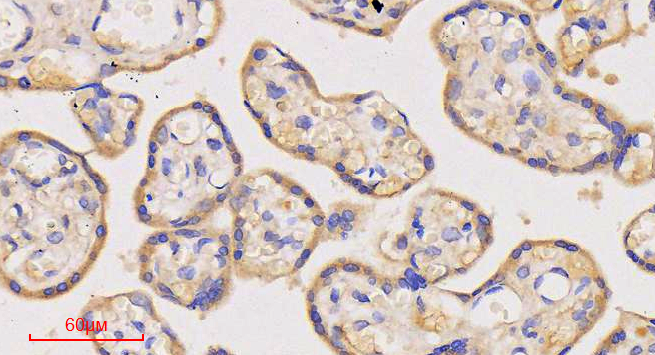

Supplement: Data S2. Original immunohistochemistry (IHC) staining images for F2R and GAL, related to Figure 7 — Uncropped, full-resolution images of all IHC stains shown in Figure 7, including both low- and high-magnification fields for FGR and AGA samples. Provided as a single PDF file. [file mmc6.zip › Data2/(FGR,GAL高表达,高倍镜x20)2132451 5_viewcapture.png]

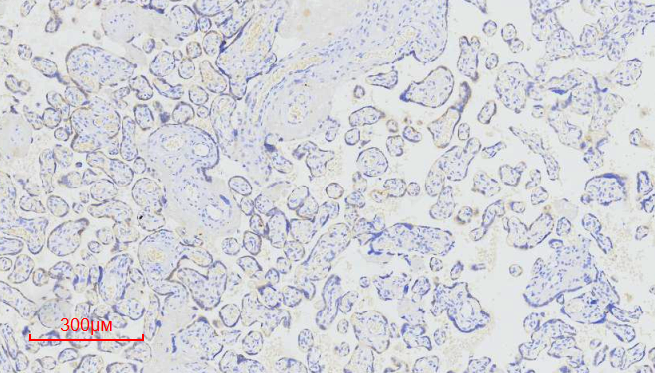

Supplement: Data S2. Original immunohistochemistry (IHC) staining images for F2R and GAL, related to Figure 7 — Uncropped, full-resolution images of all IHC stains shown in Figure 7, including both low- and high-magnification fields for FGR and AGA samples. Provided as a single PDF file. [file mmc6.zip › Data2/(正常体重,F2R 低表达,低倍镜x4)2455797 5_viewcapture.png]

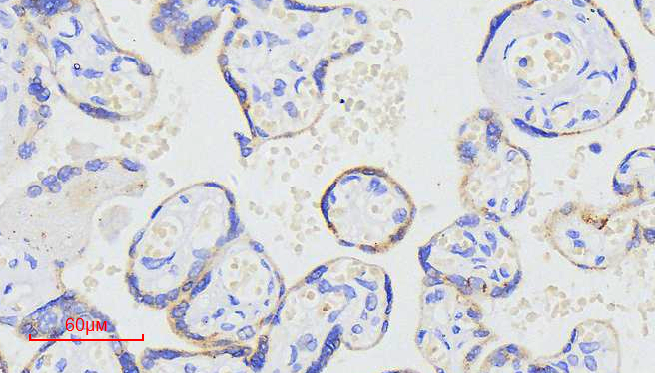

Supplement: Data S2. Original immunohistochemistry (IHC) staining images for F2R and GAL, related to Figure 7 — Uncropped, full-resolution images of all IHC stains shown in Figure 7, including both low- and high-magnification fields for FGR and AGA samples. Provided as a single PDF file. [file mmc6.zip › Data2/(正常体重,F2R 低表达,高倍镜x20)2455797 5_viewcapture.png]

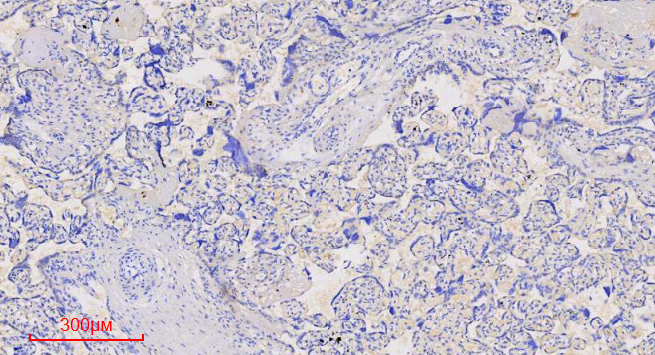

Supplement: Data S2. Original immunohistochemistry (IHC) staining images for F2R and GAL, related to Figure 7 — Uncropped, full-resolution images of all IHC stains shown in Figure 7, including both low- and high-magnification fields for FGR and AGA samples. Provided as a single PDF file. [file mmc6.zip › Data2/(正常体重,GAL低表达,低倍镜x4)2510230 5_viewcapture.png]

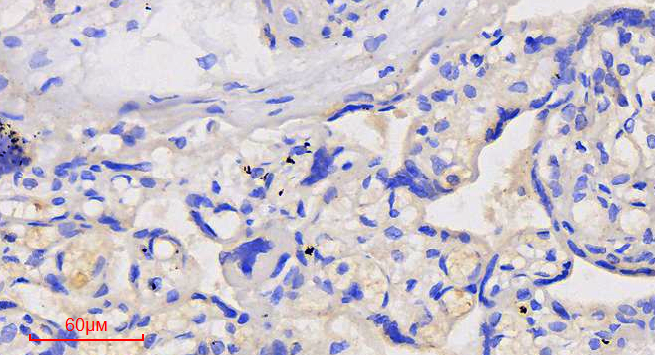

Supplement: Data S2. Original immunohistochemistry (IHC) staining images for F2R and GAL, related to Figure 7 — Uncropped, full-resolution images of all IHC stains shown in Figure 7, including both low- and high-magnification fields for FGR and AGA samples. Provided as a single PDF file. [file mmc6.zip › Data2/(正常体重,GAL低表达,高倍镜x20)2510230 5_viewcapture.png]
