# Supplementary material for: GAL and F2R as immune diagnostic biomarkers for fetal growth restriction
Source: iScience. 2026 Mar 4;29(4):115228. doi: 10.1016/j.isci.2026.115228 (PMC13015252; doi:10.1016/j.isci.2026.115228)
Supplement: Document S1. Tables S2 and S4–S6 [file mmc1.pdf]

**iScience, Volume *29***

## Supplemental information

# GAL and F2R as immune diagnostic biomarkers for fetal growth restriction

**Shiying Chen, Yumin Ke, Zhimei Zhou, Yajing Xie, Weihong Chen, Li Huang, Liying Sheng, Yueli Wang, Binbin Chen, Congmei Yang, and Zhuna Wu**

Table S2 19 IR-DEGs were found by intersecting the DEGs, and IRGs.

| |
|---|
| TNFRSF8 |
| GAL |
| IL17D |
| LGR5 |
| OPRK1 |
| IL1B |
| CXCL10 |
| F2R |
| SERPINA3 |
| CCL8 |
| ENDOU |
| CXCL9 |
| HLA-DQA1 |
| IL1R2 |
| TNC |
| NDP |
| PRL |
| CCK |
| C3 |

Table S4. Summary of Immune Cell Composition and Its Correlation with F2R and GAL

| | **Mean Proportion in FGR Group** | **Mean Proportion in AGA Group** | **P-value (Group Difference)** | **Correlation with F2R Expression** | **Correlation with GAL Expression** |
|---|---|---|---|---|---|
| B cells naive | 0.042 | 0.062 | 0.102 | -0.12 | -0.08 |
| B cells memory | 0.004 | 0.007 | 0.245 | 0.05 | 0.03 |
| Plasma cells | 0.071 | 0.068 | 0.876 | -0.18 | -0.21 |
| T cells CD8 | 0.028 | 0.019 | 0.304 | 0.22 | 0.15 |
| T cells CD4 memory resting | 0.131 | 0.149 | 0.456 | -0.31* | -0.29* |
| T cells CD4 memory activated | 0.036 | 0.039 | 0.812 | 0.41** | 0.38** |
| T cells follicular helper | 0.021 | 0.013 | 0.218 | 0.52*** | 0.49*** |
| T cells regulatory (Tregs) | 0.045 | 0.048 | 0.754 | 0.33* | 0.36** |
| T cells gamma delta | 0.032 | 0.012 | 0.042* | 0.28* | 0.31* |
| NK cells resting | 0.014 | 0.021 | 0.189 | -0.14 | -0.11 |
| NK cells activated | 0.025 | 0.012 | 0.038* | 0.19 | 0.23 |
| Monocytes | 0.034 | 0.031 | 0.672 | 0.09 | 0.12 |
| Macrophages M0 | 0.008 | 0.003 | 0.105 | 0.14 | 0.10 |
| Macrophages M1 | 0.069 | 0.035 | 0.009** | 0.58*** | 0.62*** |
| Macrophages M2 | 0.101 | 0.115 | 0.421 | -0.26* | -0.30* |
| Dendritic cells resting | 0.045 | 0.028 | 0.047* | 0.44** | 0.47*** |
| Dendritic cells activated | 0.006 | 0.002 | 0.112 | 0.08 | 0.11 |

| | Mean Proportion in FGR Group | Mean Proportion in AGA Group | P-value (Group Difference) | Correlation with F2R Expression | Correlation with GAL Expression |
|---|---|---|---|---|---|
| Mast cells resting | 0.265 | 0.312 | 0.189 | -0.36** | -0.40*** |
| Mast cells activated | 0.034 | 0.012 | 0.025* | 0.45** | 0.48*** |
| Eosinophils | 0.016 | 0.021 | 0.312 | -0.11 | -0.13 |
| Neutrophils | 0.010 | 0.011 | 0.901 | 0.25 | 0.22 |

*Note: P-values were calculated using the Mann–Whitney U test; correlations are Spearman correlation coefficients. *p < 0.05, **p < 0.01, **p < 0.001.

**Table S5.** Summary of General Information on Included Datasets

| | **Inclusion** | | **Exclusion** | **Clinical characteristics of samples** | **sample source** | **Extracted molecule** |
|---|---|---|---|---|---|---|
| | **FGR** | **Controls** | | | | |
| **GSE24129** | unexplained fetal growth restriction (n=8) | normal pregnancies (n=8) | preeclamptic (n=8) | All samples delivered by Caesarean sections | Human placenta | total RNA |
| **GSE100415** | birthweight <10th percentile for gestational age and sex based on a Canadian growth reference(n=20) | / | / | Including maternal age,ethnicity, blood type, pre-pregnancy weight, height, BMI; gestational age at delivery; placental weight; placental thickness; cord diameter; max systolic and diastolic blood pressure | Human placenta | total RNA |
| **GSE147776** | Fetuses with an estimated weight lower than the 10th percentile of the corresponding gestational age weight (ACOG, 2013 criteria) (n=7) | Normal Pregnancy(n=8) | Preeclampsia (n=7), Preeclampsia and Intrauterine Growth Restriction (n=6) | All samples had intact membranes, no clinical signs of intrauterine infection, singleton pregnancy, and were delivered by cesarean section | Human placental | total RNA |

Table S6. Summarizing placental sample collection and processing methods across the three GEO datasets

| Dataset | Study (Year) | Biopsy Method | Sampling | Processing | RNA Extraction | Key Feature |
|---|---|---|---|---|---|---|
| **GSE 24129** | Nishizawa et al. (2011) | Central chorionic tissue collected after cesarean section, avoiding decidua or amnion | Four 1 cm sections from different central areas between basal and chorionic plates | Washed with saline to remove maternal blood, then snap-frozen in liquid nitrogen | RNeasy mini-kit (Qiagen) | Only samples from women without labor to exclude labor-associated gene expression changes |
| **GSE 100415** | Gibbs et al. (2018) | One biopsy per placental quadrant midway between cord insertion and disc periphery | Biopsies from the four quadrants were pooled | Rinsed in PBS, snap-frozen, and ground into powder | Trizol + RNAeasy spin columns | Samples from normotensive SGA pregnancies with suspected FGR, all delivered >34 weeks gestation |
| **GSE 147776** | Medina-Bastidas et al. (2022) | Full-thickness tissue collected immediately after cesarean section from the central placental region, near cord insertion, avoiding decidual/amnionic areas | Tissue blocks collected from this region to ensure representativeness | Washed with cold 1× RBC lysis buffer and 1× PBS to remove maternal blood contamination | RNeasy Fibrous Tissue Mini Kit (Qiagen) | All samples from cesarean deliveries without signs of labor to exclude effects of labor-related gene expression changes |
